# A qualitative exploration of mental health knowledge among pediatric health professionals in the United Arab Emirates

**DOI:** 10.1371/journal.pone.0266224

**Published:** 2022-03-29

**Authors:** Nabeel Al-Yateem, Rachel Rossiter, Muhammad Arsyad Subu, Shameran Slewa-Younan, Syed Azizur Rahman, Jacqueline Maria Dias, Amina Al-Marzouqi

**Affiliations:** 1 Department of Nursing, College of Health Sciences, University of Sharjah, Sharjah, UAE; 2 School of Nursing, Midwifery and Indigenous Health, Faculty of Science, Charles Sturt University, Bathurst, NSW, Australia; 3 School of Medicine, Western Sydney University, Campbelltown, Australia; 4 Department of Health Services Administration, College of Health Sciences, University of Sharjah, Sharjah, UAE; 5 Department of Health Services Administration, University of Sharjah, Sharjah, UAE; Prince Sattam Bin Abdulaziz University, College of Applied Medical Sciences, SAUDI ARABIA

## Abstract

**Background:**

Mental health literacy (MHL) is an essential competency for all healthcare professionals. In the United Arab Emirates, previous studies reported a low level of MHL among healthcare professionals working with vulnerable populations such as children and adolescents with chronic illnesses. Further in-depth exploration is necessary to build understanding of beliefs and knowledge about mental illness among pediatric health professionals.

**Methods:**

Written narratives exploring mental health knowledge were collected from pediatric nurses and analyzed using content analysis. These written narratives were extracted from responses to open-ended questions embedded in a questionnaire completed as part of previous studies. The Standards for Reporting Qualitative Research were followed in reporting this study.

**Results:**

The overarching theme that emerged from the data was that nurses struggled to negotiate the complexities of psychological distress and mental illness. Two overlapping sub-themes were identified: (1) professional knowledge was incomplete, confused, and lacking in clarity and (2) professional knowledge was impacted by cultural beliefs and stigma. A third sub-theme reflected how participants identified with others’ suffering and felt powerless to help themselves or others. Participants described stress and mental exhaustion.

**Conclusions:**

Participants’ narratives were characterized by struggles arising from insufficient knowledge, confusion, and deeply-held cultural and religious beliefs. Therefore, they were unable to resolve the conflict between professional knowledge, attitudes, and beliefs about mental illness and stigma arising from cultural and religious beliefs/attitudes. Culturally-specific education is needed for healthcare professionals that addresses contextual, cultural, and religious factors impacting on stigma while actively supporting the healthcare workforce and enabling access to mental health services.

## Introduction and background

Health literacy refers to people’s knowledge, motivation, and competence in accessing, understanding, appraising, and applying health information. High health literacy has been linked to good judgment and decision-making concerning healthcare, disease prevention, health promotion, and maintaining or improving quality of life throughout the life course [1]. Conversely, low levels of health literacy have been linked to a range of adverse health impacts and increased costs as a result of avoidable healthcare and hospitalization [1, 2]. Although the importance of improving health literacy is widely accepted and has been the focus of various interventions around the world, a similar level of attention has not been directed to improving mental health literacy (MHL) [2–4]. MHL has been defined as “knowledge and beliefs about mental disorders which aid their recognition, management or prevention.” It also “includes the ability to recognise specific disorders; knowing how to seek mental health information; knowledge of risk factors and causes, of self-treatments, and of professional help available; and attitudes that promote recognition and appropriate help-seeking” [5].

Current international evidence has identified deficits in MHL, along with stigmatizing beliefs and negative attitudes about people experiencing mental health issues that originate from local traditional cultures and religions. For example, some cultures and religions believe that demons or spirits can control of a person’s body; such possession is believed to cause mental illness, which therefore requires shamanic treatment [6–10]. In some countries where these beliefs prevail, treatment for much of the population (i.e., rural and traditional communities) has mainly focused on traditional and religious treatments. Strongly-held stigmatizing beliefs affect patients and their family members and give rise to feelings of shame, embarrassment, social isolation, and guilt. Access to treatment, recovery from illness, and rehabilitation have also been reported to be affected by stigma in communities. Conversely, in environments with low levels of stigmatizing beliefs, people with mental illness and their families can access good social support networks and therefore recover more easily and develop better coping skills [6–12]. In the United Arab Emirates (UAE), Gulf Cooperation Council region, and the Middle East in general, stigmatizing attitudes and beliefs have been found among the general public and healthcare professionals, including those working with vulnerable populations such as children and adolescents with long-term health conditions [10, 13–17].

Awareness of mental health issues is essential for healthcare professionals caring for children and adolescents with chronic and life-limiting health conditions. A range of studies have reported these illnesses have negative effects on adolescent health, development, and the transition to adulthood. Mental health problems and disrupted development may also occur following repeated hospitalization, ongoing periods of poor health, decreased physical strength and skills, and changes in appearance due to illness [18–20].

In the UAE, a substantial proportion of the population are young people. The developmental needs of this population require effective physical health services and evidence-based psychological services that support them as they grow toward adulthood. Furthermore, the UAE has a high prevalence of chronic conditions such as diabetes, asthma, and cardiovascular diseases, which are currently ranked as the top causes of death and disability among all age groups [21]. The need for an increased focus on mental health services for children and young adults is supported by research evidence that young people experiencing psychological and emotional distress indicative of emerging mental health problems were less likely to seek professional help or be referred to mental health services than older people. In addition, the prevalence of psychiatric disorders among UAE children was reported as 23.9%, which was similar to rates reported in studies from Western countries [22–29]. Finally, recent evidence suggests that MHL among UAE healthcare professionals working with this population is low and needs urgent attention [13, 14].

Marked progress has been made in developing quality health services in the UAE; however, mental health services and healthcare professionals require focused training and professional development to challenge existing levels of stigma and improve MHL [13, 14, 22, 23, 25, 26, 29]. The UAE Ministry of Health and Prevention has identified mental health as among the country’s top five health priorities [30]. The UAE Government’s 2020 vision specifies the upgrade of healthcare services to match levels in developed countries as a main focus area [31].

This study reports on the qualitative component of two studies conducted in the UAE that explored MHL among healthcare professionals working with children and adolescents with chronic conditions in school- or hospital-based contexts [13, 14]. Both studies used a culturally-adapted MHL questionnaire and a cross-sectional survey design to measure mental health knowledge among healthcare professionals (total number of participants across the two studies = 718) working with child and adolescent populations. The questionnaire presented participants with vignettes of patients with three common mental health conditions (i.e., posttraumatic stress disorder, depression with suicidal thoughts, and psychosis). Participants were assessed based on their answers to closed-ended multiple choice or ranking questions related to diagnosis and treatment options for the patients in the vignettes. The questionnaire also included open-ended questions, the responses to which have not previously been reported.

Analysis of the quantitative data from these two studies [13, 14] demonstrated a problematic level of MHL among participating healthcare professionals, especially in the recognition of mental illnesses and beliefs about treatment for these conditions. Only 49% of school-based and 54% of hospital-based healthcare professionals who worked with child and adolescent patients were able to correctly recognize the existing mental health problems experienced by the patients in the vignettes [13, 14]. This analysis was important in identifying key aspects of MHL (e.g., identification and treatment knowledge) among respondents; however, a deep understanding of healthcare professionals’ experiences and beliefs is equally important and requires exploration using a qualitative inquiry. A review of the literature highlighted the absence of such qualitative studies, especially in the UAE and local region. Most available literature about MHL is from the United States, Australia, Canada, and Europe. In addition, most previous researchers studied MHL from public or general population perspectives [13–16, 32–45]. Similar studies focusing on healthcare professionals are scarce, particularly studies using qualitative methodologies. The present paper reports the findings of a qualitative analysis of participants’ written answers to the open-ended questions in the questionnaires. Given the low levels of MHL revealed by quantitative data in the two previous studies [13, 14], analysis of responses to the open-ended questions in those surveys offers opportunity to enhance understanding of possible contributors to healthcare professionals’ knowledge and beliefs. A qualitative perspective of MHL among this population will provide information to inform future planning for prevention, promotion, management, and development of mental health programs for healthcare professionals in the UAE that are fit for purpose.

### Study aim

This study aimed to conduct an in-depth exploration of MHL responses from pediatric health professionals in the UAE, build an understanding of their beliefs and knowledge about mental illness, and provide information to inform future planning of mental health programs in the UAE.

## Methods

### Context and participants

This study was conducted at three major hospitals and school clinics that provide care for children with chronic illnesses in different UAE Emirates (i.e., cities). The study sites represented both acute and primary care settings. To ensure the sample was representative, different Emirates were included that had different healthcare services and models of healthcare delivery. The UAE healthcare system comprises a federal governing authority that runs public healthcare services (i.e., Ministry of Health and Prevention) and local healthcare authorities that run their own healthcare services following their own models of care.

The main hospital and clinical settings where participants worked were purposefully selected and approached through in-person meetings and virtual/telephone communications to seek approval for their staff to participate in this study. Once approval was obtained, a paper-based survey was distributed to potential participants by these authorities. Those who were willing to take part in this study completed and returned the questionnaire as per the instructions provided. Two reminders were sent to healthcare professionals encouraging them to take part in this study and return their completed questionnaire.

Across these different settings, 656 health professionals working with children and adolescents with chronic conditions completed the questionnaires. These 656 participants included 339 school nurses employed in government and private schools from three Emirates and 317 healthcare professionals from major UAE hospitals treating pediatric populations.

### Study design and qualitative approach

Data were collected in a cross-sectional questionnaire that included both closed- and open-ended questions. This paper used a qualitative approach to analyze participants’ responses to the open-ended questions (written narratives). The questionnaire contained three vignettes discussing patients with common mental health conditions (i.e., posttraumatic stress disorder [PTSD], depression with suicidal thoughts, and psychosis) (S1 Appendix). Participants were asked to provide written responses to open-ended questions covering: knowledge, attitudes, and beliefs related to patients with mental health problems; possible discrimination that these patients may experience; helpful and harmful treatments or medicines that may be offered to these patients; health professionals or community groups that may be helpful resources for these patients; the seriousness and difficulty of treating mental health conditions; and their experience of dealing with people or patients with mental health difficulties.

### Data collection and analysis

A paper-based survey was distributed to participants after permission was granted by relevant health services and authorities. Based on available data about the study population and number of surveys distributed and returned, the response rate was estimated as 35%–60%. The questionnaire was presented in English, after adaptation to suit the local context and validation against the Manual for Assessment and Diagnosis of Mental Disorders, Fifth Edition diagnostic criteria for the included clinical vignettes. The questionnaire is a well-known and validated instrument [5, 38], and was further piloted with 10 participants with the same cross-cultural backgrounds/languages as the target population (e.g., Arabic, Indian, Filipino).

The use of open-ended questions as a component of the overall questionnaire enabled participants to share maximum information while also protecting their anonymity. This approach is known to improve data sharing by participants, especially for sensitive topics such as that explored in this study. Examples of the open-ended questions asked in the survey are:

1. Imagine X (patient name in vignette) is someone you have known for a long time and care about. You want to help her. What would you do?
2. Can you please give examples of ways you think X would be discriminated against?
3. What do you think is most likely to be the greatest risk factor for somebody developing a problem like X?

The qualitative approach used to analyze participants’ responses was the content analysis method first proposed by Schreier in 2012 [46]. Content analysis is an interpretive process that focuses on exploring the similarities and differences between and within different parts of participants’ narratives, enabling an in-depth understanding of participants’ knowledge, attitudes, and beliefs about the issue of interest [46]. Participants’ written narratives were extracted from the data collection sheets and placed in a separate text file. Codes and units of meaning were interpreted in the context of the study focus and compared for similarities and differences. The emerging results were then discussed among the study team to construct major themes. Of the 656 completed questionnaires, only those that contained adequate elaboration in the open-ended questions were included in the qualitative content analysis.

To gain an in-depth understanding of and analyze participants’ narratives, the research team met and consulted regularly throughout the analysis process and when preparing the report on the findings. The team discussed the data and reviewed the analysis to edit the emerging categories and themes as necessary (i.e., second level analysis). When new understanding was reached, it was discussed and deviations from the word-for-word coding process and initial data analysis were assessed to ensure that changes to the findings and interpretations were valid and did not contradict the initial interpretation, but rather developed and deepened the interpretation.

### Ethics approval and consent to participate

Ethical approval was obtained from the Research Ethics Committees of the University of Sharjah (Ref#:ERC/23/11/15/46), the Dubai Scientific Research Ethics Committee (Ref#: DSREC-12/2015_13), and the Ministry of Health and Prevention Research Ethics Committee (Ref#: R04). Written informed consent was obtained from all participants. Full anonymity was ensured during this study, and no personal or identifying information was collected as part of this study.

## Results

All 32 healthcare professionals whose written narratives were analyzed were nurses that worked with children and adolescents in either the hospital setting or school clinics in the UAE. Participants were aged 23–52 years and had a minimum clinical experience of 3 years.

### Thematic analysis

The analysis of participants’ narratives revealed that nurses struggled to negotiate the complexities of psychological distress and mental illness. These struggles were represented by three themes: 1) Professional knowledge was incomplete, confused, and lacking clarity; 2) Professional knowledge was impacted by cultural beliefs and stigma; 3) and Identifying with suffering and feeling powerless to help themselves or others.

### Theme 1: Professional knowledge was incomplete, confused, and lacking in clarity

Participants’ attempts to describe their knowledge of the mental illnesses described in the vignettes were incomplete, confused, lacking clarity, and inconsistent with evidence-based understanding. Many participants were unable to accurately recognize the type of mental health problem experienced by the patients in the vignettes and could not determine the type of diagnoses that could be offered. They were not able to elaborate on clear, complete, and evidenced-based treatment or support for the mental health problems demonstrated in the vignettes. Although participants could recognize that the patient was experiencing issues, they could not determine if the problem was medically significant or merited further intervention. For example, one participant reported:

“I don’t recognize and understand M’s problem (PTSD), frankly. What is it? Is it depression? Or…maybe stress, not sure…maybe it is just a transient breakdown. I can’t tell for sure.” (Participant 3; Female)

Another participant said:

“…I don’t exactly know what is A’s main problem (vignette character with depression with suicidal thoughts). I do not know…I am not familiar. Sorry, I don’t know, I am not sure about it. I should learn mental illness.” (Participant 11, Female)

Similarly, participants were unable to consistently demonstrate a sound understanding of the risk factors for mental illness or population groups that were particularly vulnerable to mental health problems. Some participants correctly identified some risk factors for mental health problems such as having complex health issues, family problems, and coming from a war-torn country. Other participants identified people who were unemployed, poor, or older as more vulnerable to mental illness. One participant wrote:

“The unemployed, the poor, very sick people, especially children and older people, they are easier to have mental illness. Probably because of the many problems that they had in life.” (Participant 16, Female)

Participants described religion as a protective factor against mental illness and noted a lack of active engagement in religious practices as a potential risk factor. For example, one participant commented:

“One important thing that protects you from having mental illness is reading [the] Koran, praying, and similar stuff. This will protect you and strengthen you. Not doing so, the person will be vulnerable to mental problems.” (Participant 1, Female)

Another participant’s reflection on risk factors for mental illness identified the value of a supportive family:

“I am not sure but…you know, being born in a war-torn country is the greatest risk factor of mental health. Also, family members and friends play an important role in supporting a mentally ill [person]. I believe…People who have families are not likely to have mental illness.” (Participant 6, Female)

Participants’ narratives indicated they were confused about available treatments and supportive interventions for mental illness. Some participants described useful treatment options such as seeking social support, religious and spiritual practices, and visiting specialized mental health services. However, in many instances, participants thought religious and traditional treatment options were equally or more useful than psychiatric services. Many participants described reading the Koran and praying as the most helpful treatments, and noted that use of psychiatric drugs required caution. One participant wrote:

“The most helpful is reading Koran, reciting ‘athkar’ (spoken words recited by the person in remembrance of Allah/God as a form of worship) and prayers, especially reading it with a religious leader. Very…very…very helpful. Also doing prayer five times a day is important. This of course along with seeing a specialized doctor, although it is not easy for people to take their patients to a psychiatric doctor…No, they will feel shy from friends and relatives.” (Participant 21, Female)

Another participant reflected on the most helpful treatment offered for patients:

“People use so many traditional therapies that they think helpful. Some herbs, honey, olive oil, dates, and cupping are available that [are] most helpful for the patient. I understand that, especially in our culture.” (Participant 7, Male)

Non-professional help-seeking appeared to be preferred over professional sources of mental healthcare. Most responses identified non-healthcare professionals as the first source of help. Participants suggested “a close family member, or a close female or male friend first, as they will be the most helpful people to take care of them.” Another participant indicated that:

“Family members or friends are helpful resources relating to mental health problems. Talking or [being] open to discuss the problem to their family members is helpful. Otherwise, a close friend is also helpful for patient with mental health issues. They are important persons to help…open discussion is important.” (Participant 10, Female)

Few participants suggested that the first option for help-seeking should be a specialized mental health professional; these participants reported that they would recommend the patient visit a “psychiatrist,” “psychologist,” “local doctor,” or “a community health worker/team.”

Mental illness was commonly viewed as hereditary in nature or difficult to treat/untreatable. Some participants labeled mental illnesses as purely hereditary illnesses; once one person in the family has such an illness, others are at high risk. One participant described the hereditary nature of the illness, as follows.

“Yes, it is true. It runs in family…it is genetic illness or from generation to generation, inherited. Patients with mental illness have a parent or parents with mental illness. [If] a parent has a mental illness, their children will have mental health issue.” (Participant 4, Female)

Another participant’s response clearly demonstrated they believed the patient was likely to have a poor outcome:

“I think the likely result if S (vignette character with psychosis) received the sort of help, it is only partial recovery, but…his mental health problem will re-occur or will get worse in the future.” (Participant 18, Male)

### Theme 2: Professional knowledge was impacted by cultural beliefs and stigma

This second theme overlapped with the perspectives reported under the first theme. The researchers anticipated that participants’ professional knowledge and training as nurses would moderate responses towards the narratives describing a person with a mental illness. However, this was not always apparent. The beliefs and attitudes participants expressed were often similar to those commonly found in the local culture and community. Some participants described the cause of mental illness was a person’s “weak character” or that they were “not sufficiently religious.” Other participants could correctly identify some causes or contributors of mental health problems, such as “extra anxiety or pressure from illness or work,” “coming from a war-torn country,” or “being separated from family or loved ones.” However, a pervasive belief in an inherent weakness was apparent, as even when participants correctly identified factors contributing to mental illness, their narratives expressed a belief that the person must be strong and overcome their circumstances with the help of their religion, family, or friends.

“All people have their own issues, it is life…stress, stress, stress. You need to get over it and move on…I believe that M’s (vignette character with PTSD) main problem is a weak character.” (Participant 23, Female)

Some narratives demonstrated empathy for the person experiencing mental illness, acknowledging these problems as difficult to live with and treat. For example, one participant wrote:

“Yes, it (a mental health problem) is quite distressing. Counseling with mental health professionals can help A (vignette character with depression with suicidal thoughts), I think for A’s problem it is difficult to treat…Not easy at all.” (Participant 19, Female)

In contrast, others expressed sympathy for the patient, but suggested that the problem was continuing because the person was weak and not seeking religious and family support.

“In these situations, you need to be strong, not weak. She (vignette character with PTSD) must be trying to deal with the problem on her own. Have a prayer session or reading with a religious leader. Reading the Koran or Bible. Talking about the problem with a family member or maybe a close friend.” (Participant 12, Female)

Widely differing perspectives were also evident when participants wrote about the best treatments for mental health problems. Some participants believed that approaching religious leaders or adopting religious practices should be among the first options for patients, which if unsuccessful, could be followed with seeking access to healthcare or psychiatric services. For example:

“Yes, it is true in this situation. If mental health issue that experienced by M (vignette character with PTSD), religious leader, community religious organization…These are good to approach first for help. I also believe these people can help M to deal with her problem. These people are important for mental health problems in community. Reading the Koran is also helpful, I believe.” (Participant 5, Female)

Despite advocating for family and friends as first sources of assistance, the perceived stigma associated with accessing psychiatric services implied that doing so would adversely impact the whole family.

“Family…friends or both. Family members and close friends are important to approach first in this case. You know, mental illness…I will talk to my family and my close friend first…I will talk to them to get help with my problem first. Is it true right? They are important people in my life to help me. Going to a psychiatrist is not an easy thing in our culture, the whole family will suffer.” (Participant 13, Female)

Although they strongly favored cultural, religious, and family support for people with mental illness, participants were able to identify harmful substances or coping strategies some people used to manage symptoms of mental illness. These included use of alcohol and illicit drugs, and non-evidence-based treatments such as hypnosis. Participants indicated that patients needed support to avoid harmful substances or withdraw from substances. For example, one participant reported:

“Many (patients) use a treatment that harmful for them…For example, drinking alcohol to relax or to solve the problem. However…the patient does not solve the problem but creates another problem. They need to know that alcohol consumption is harmful as a mental health treatment.” (Participant 20, Female)

Ambivalence towards commonly prescribed antipsychotic medicines was apparent, with one participant expressing a perspective that antipsychotics were both “noxious” and unlikely to be beneficial.

“Oh…these medications are horrible. Not effective with many patients…it has so many negative symptoms and side effects…it can be very noxious, you know, I don’t like it, maybe with severe cases, but for me I don’t like it.” (Participant 8, Female)

Overall, participants’ narratives displayed awareness of the stigma and discrimination that people with mental illness experienced as they struggled to manage their condition and seek treatment. This was one of the few issues where consensus was apparent. They also described concerning issues that people living with mental illness encountered, including rejection and isolation arising from widespread cultural beliefs. These included stigmatizing beliefs such as demonic possession, the evil eye, and being punished by God.

### Theme 3: Identifying with suffering and feeling powerless to help themselves or others

The third theme that emerged from the data revealed that some participants clearly identified with the suffering they observed in people living with mental illness, and some could relate this to their own experiences. Perception of a limited capacity to assist in a meaningful way added to their difficulties and gave rise to a feeling of being powerless to assist. Many participants reported recent examples in their own lives when they had felt depressed and sad, like the vignette characters. They reported experiencing feelings of nervousness and restlessness, and often feeling tired without reason. Perhaps, unexpectedly, they identified with the patients in the vignettes. For example, some participants described similar backgrounds in terms of having left their own home in a war-torn country, been witness to traumatic events, and separation from their families. Other participants described experiencing intense and overwhelming stress. One participant wrote:

“You ask me if I feel similar sometimes, yes, it happens many times in my life. I feel tired out for no good reason…sometimes or often. I am sure that sometimes, I have a problem like M (vignette character with PTSD).” (Participant 14, Male)

Another participant reported:

“I feel similar for sure. I have family in (name of war-torn country) that I have not seen for long time. I am so worried about them. I contact them as much as I can, sometimes during my work. But, you know, you can’t stop thinking about them” (Participant 15, Female)

Some participants described having approached other people for help and support on issues such as those experienced by vignette patients. Examples of people approached included family members, friends, and (less often) general practitioners in primary healthcare centers. One participant wrote:

“I had a lot of pressure and stress recently that I called my friend X and spoke for a long time…I felt a little better afterward…but the stress comes back again and again. Some nights I can’t sleep…that night I went to the health center close to me for help.” (Participant 17, Female)

Another participant described the distress as overwhelming:

“Many times, I take medication to sleep, I can’t stop thinking of some issues related to my work and family back home. I feel my head will be blown away…at the end I take the tablet hoping that I will fall asleep.” (Participant 9, Female)

Despite sometimes feeling under enormous pressure and experiencing stress and difficulty, participants tended to seek help from close friends, avoided colleagues, and did not seek any professional help. Several reasons for avoiding professional help were offered. For example:

“Oh no, I don’t tell anyone. I try to look normal at work. I want to keep my job. I can’t disclose to work colleagues or go to a doctor. No. Doctors are also expensive I can’t afford.” (Participant 22, Female)

They also reported feeling sorry, worthless, and powerless because they were not able or felt inadequate to offer therapeutic help and support for their patients.

“I feel sad when I can’t help patients like A (vignette character with depression with suicidal thoughts). We don’t have support system for somebody like him…we don’t even have a policy about what to do for them. Many times, I had suspicions but did not know what to do. That is stressful…and so many times you just need to suppress your feeling and carry on…but it does not feel right.” (Participant 25, Female)

## Discussion

To our knowledge, this was the first qualitative study to explore MHL among nurses working with vulnerable populations (i.e., children and adolescents with chronic conditions) in the UAE. Our findings contribute to the few international studies in this area. It was postulated that a qualitative design would enable an in-depth understanding of key contributors that influenced MHL among healthcare professionals in the UAE [13, 14]. The present findings supported the results of previous quantitative studies that were conducted in the UAE, along with international studies that assessed healthcare professionals’ knowledge about mental illnesses, risk factors, diagnosis, and treatment. Those studies reported problematic levels of MHL among healthcare professionals (including nurses) that had potential to compromise their ability to offer adequate help for their patients [4, 13, 14, 47, 48].

Our findings revealed three primary themes. The first theme was characterized by a high level of confusion and lack of clarity among participating healthcare professionals in terms of their knowledge about identifying, treating, and dealing with patients at risk for or with mental health problems. Our findings demonstrated that some participants had partially correct information about the causes of mental illness and interventions available for patients. However, this information was limited, and usually coupled with other inaccurate information. For example, participants acknowledged that extra pressure or anxiety (as a result of factors such as illness, life stressors, and peer pressure) may be a reason for developing a mental illness. However, they also noted that the patient might have had a weak character that made them vulnerable to mental illness or might have been a victim of an evil eye that caused the problem.

An important finding was the dissonance apparent between cultural beliefs expressed by participants and the medical and nursing knowledge and research evidence that they had learned as part of their professional education. This dissonance could be described as a pervasive challenge for nurses in the UAE, and one that may compromise the care that they and other healthcare professionals are able to both initiate and provide. Potential explanations for this dissonance include the strength of the pervasive cultural beliefs regarding mental illness, including believing in phenomena such as the evil eye (curse inflicted by a malevolent gaze), demonic possession, and other religious beliefs. These cultural beliefs combined with limited undergraduate coverage of mental illness has potential to mean health professionals lack confidence in their ability to assist people that demonstrate signs and symptoms of mental illness.

Previous studies conducted in Switzerland and Australia found that healthcare professionals showed high levels of MHL [49], demonstrated by high levels of recognition of mental illness such as schizophrenia and depression [50]. However, these studies noted that increased levels of recognition did not equate to decreased stigmatization by professionals about patients with mental illness or some mental health treatments, or increase their willingness to interact with these patients [49, 50]. This highlights the complexity of stigma related to mental illness and possible contributing factors. Healthcare professionals’ cultural and religious beliefs also influence their clinical choices, decisions, and practice. A qualitative study that explored the impact of South African Muslim general practitioners’ beliefs about mental health on their clinical practice highlighted the need for greater awareness of mental illness among these healthcare professionals, along with more understanding of the differing religious and cultural taxonomies of illness held by the people to whom they provided care [36, 51]. It was concerning to see the extent to which these cultural beliefs conflicted with or were incompatible with medical evidence and nursing knowledge about mental illness. Participants’ narratives indicated that these nurses held cultural beliefs attributing mental illness to causes such as a weak character, punishment from God, failure to actively engage with the Koran, or dwelling on problems rather than moving on. Importantly, participants’ cultural beliefs seemed to take precedence over their professional training, with some narratives directing blame towards the mentally ill person. Participants’ narratives also indicated awareness of the stigma and discrimination that people with mental health issues may face in their local community or when seeking healthcare services. Participants also reported more serious issues that people experiencing mental illness may face, such as “rejection,” “isolation,” and being described as having “demonic possession,” the “evil eye,” or “being punished by God.” For improvement in the care and treatment of people experiencing mental illness to occur, focused attention is required to increase MHL in the nursing workforce and in the broader community.

Finally, a key finding of this qualitative analysis was the way in which the participants (i.e., nurses) described their own levels of stress and distress and the barriers to them engaging in help seeking. These findings were consistent with the statistical findings reported in the previous quantitative studies [13, 14], which found that a large proportion (68%) of school healthcare professionals had high or very high psychological distress, 24% had moderate distress, and 32% mild levels of distress. In addition, of the 317 hospital-based healthcare professional participants, almost 40% had a stress level in the mid-to-high-range. From the perspective of practical implications, this could be considered the most important finding as it indicates that targeted interventions/campaigns for these professional is needed. These interventions seem to be a pre-requisite and foundation for further work to achieve meaningful and real development. Given the importance of this issue, specific communication was implemented with the managers of participating school healthcare services to highlight this finding and the researchers’ recommendations.

This study had a number of strengths. Specifically, the analysis of written responses to the open-ended questions offered participants an anonymous opportunity to reflect on their personal experiences, beliefs, and knowledge about this sensitive issue, and therefore provided significant and compelling findings. However, a significant limitation was that the use of a questionnaire precluded the possibility of an in-depth exploration of participants’ views and experiences in instances where further elaboration was needed or a point was unclear.

## Conclusions

The main characteristics of healthcare professionals’ mental health knowledge that emerged from this study are that such knowledge is incomplete, confused, and lacked clarity. When talking about the different risk factors for mental illnesses, treatment approaches, and seriousness, participants reported both correct and incorrect knowledge. However, most were confused and unsure. Furthermore, participating healthcare professionals appeared to be unable to resolve the conflict between their professional knowledge, attitudes, and beliefs in relation to mental health illness and those held by their culture and religion. Finally, healthcare professionals noted they also felt stressed and mentally exhausted, similar to the patients presented in the vignettes; this may be attributed to personal and professional difficulties that they had experienced. Participants indicated they often felt powerless as they were not able to help vulnerable patients because of personal or systemic shortcomings, which highlights the need to raise the MHL of this population.

### Study implications

This study is the first in the UAE to seek to comprehensively understand the level of mental health knowledge and beliefs among pediatric healthcare professionals. Our findings provide compelling evidence to support the design and delivery of MHL campaigns for current and future healthcare professionals. The required educational interventions should be culturally appropriate and enable clinicians to understand the impact of their personal beliefs about mental illness on their practice and encourage them to make efforts to address this issue.

The evidence that emerged in this study about the psychological distress experienced by healthcare professionals themselves raised an important issue. It signals the need to review the work environment for healthcare professionals and provide adequate support to help them overcome their difficulties. Consideration of implementing clinical supervision and screening processes is also imperative to ensure that the services provided for the vulnerable pediatric patient populations are comprehensive and delivered by competent and well supported clinical staff.

## Supporting information

S1 Appendix Original questionnaire vignettes. (DOCX)
