# Supplementary material for: A qualitative exploration of mental health knowledge among pediatric health professionals in the United Arab Emirates
Source: PLoS One. 2022 Mar 29;17(3):e0266224. doi: 10.1371/journal.pone.0266224 (PMC8963574; doi:10.1371/journal.pone.0266224)
Supplement: S1 Appendix — (DOCX) [file pone.0266224.s001.docx]

Appendix 1: Original questionnaire vignettes

Vignette 1: PTSD scenario

Miriam is a 37-year-old married woman with three children, a daughter aged 7, and two sons, aged 5 and 3. Miriam has been living in Sharjah for the past year where she works to provide an income and education for her family. She has attended her local doctor on several occasions with the primary complaint of an inability to sleep. The problem with her sleep started just before she arrived in Sharjah. Prior to leaving her homeland, Miriam witnessed much bloodshed, the complete destruction of her home, and the killing of her eldest child. Miriam often felt intense fear for her life and overwhelming helplessness. Her husband is still in their homeland looking after his parents. She reports constant nightmares in which images of death and killing disturb her sleep. She avoids talking about what she has seen and watching news channels in case she see more about homes being destroyed and people being murdered. She is easily startled when she hears loud sounds such as a car backfiring or fireworks. She has very little interest in things around her, including her children’s lives and feels little affection toward them. Finally, when questioned on how she viewed her future and plans for her life, Miriam replies that she does not have a future and doesn’t believe she will live a long life.

Vignette 2: Depression with suicidal thoughts scenario

Abdul is 30 years old. He has been feeling unusually sad and miserable for the last few weeks. Even though he is tired all the time, he has trouble sleeping nearly every night. Abdul doesn’t feel like eating and has lost weight. He can’t keep his mind on his work and puts off making decisions. Even day-to-day tasks seem too much for him. This has come to the attention of his boss, who has threatened to terminate him. Abdul feels he will never be happy again and believes his family would be better off without him. Abdul has been so desperate, he has been thinking of ways to end his life.

Vignette 3: Psychosis scenario

Saed is 24 years old and lives at home with his parents. He has had a few temporary jobs since finishing school but is now unemployed. Over the last 6 months he has stopped seeing his friends and has begun locking himself in his bedroom and refusing to eat with the family or to have a bath. His parents also hear him walking about his bedroom at night while they are in bed. Even though they know he is alone, they have heard him shouting and arguing as if someone else is there. When they try to encourage him to do more things, he whispers that he won’t leave home because he is being spied upon by the neighbor. They realize he is not taking drugs because he never sees anyone or goes anywhere.
